# Generation of tumor-infiltrating lymphocytes from pancreatic cancer lesions for cellular therapy

**DOI:** 10.1186/2051-1426-2-S3-P26

**Published:** 2014-11-06

**Authors:** Qingda Meng, Elena B Rangelova, Liu zhenjiang, Thomas Poiret, Bartek Jiri, Caroline Verbeke, Ernest Dodoo, Ralf Segersvärd, Markus Maeurer

**Affiliations:** 1 Karolinska Insitutet, Stockholm, Sweden; 2 Karolinska Insitutet, Karolinska University Hospital, Stockholm, Sweden; 3 Dept. of Neurosurgery, Karolinska University Hospital, Sweden

## Purpose

The generation of T lymphocytes with specific reactivity against autologous tumor is a prerequisite for effective adoptive transfer therapies. Pancreatic cancer-specific lymphocyte cultures from tumor infiltrating lymphocytes (TILs) may represent a viable source of T cells for the biological therapy for patients with pancreatic cancer.

## Methods

Pancreatic cancer tissue was obtained either by surgery or from biopsy specimens from 16 patients and cultured with cytokines (IL-2, IL-15and IL-21). TIL were expanded using OKT-3 and irradiated allogeneic peripheral blood mononuclear cells (PBMCs). TIL reactivity was gauged for recognition of molecularly defined tumor-associated antigens (TAAs, mesothelin, survivin and NY-ESO-1) by IFNgamma production and intracellular cytokine production (ICS). TCR VB T cell populations were tested by a panel of TCR Vb specific antibodies, along with T cell differentiation and exhaustion markers by flow cytometry.

## Results

TIL from 16/16 patients, up to 10e11 cells, could be successfully expanded using IL-2/15/21. 4 week TIL cultures showed up to 90% CD8+ T cells, yet 1/16 TIL cultures exhibited exclusively CD4+ TIL with a CD45RA-CCR7+ phenotype. 12 / 16 of TILs showed preferential expansion of TCR VB families, i.e. 99.3% in Vb13.2 in CD8+ TIL, 77% in VB1, 68.7% in VB22, 64% in VB14 for individual patients. Even biopsy specimens (about 10 mg), yielded at least 1.5 x10e9 CD8 TIL. ICS analysis showed a low frequency (up to 2.5%) of mesothelin, survivin or NY-ESO-1 reactive CD8+ TIL. TIL from a 1/16 patients showed up to 10% NY-ESO-1 specific IFNgamma and TNFalpha production in CD4+ and CD8+ T cells. Tumors from these patients are currently sequenced for mutations and subsequent testing for TIL recognition.

## Conclusion

We have optimized methods for the robust and fast generation of TIL from pancreatic cancer lesions, including small biopsy specimens, using a cytokine cocktail of IL-2/IL-15 and IL-21. TIL showed a Th1-cytokine production pattern and a central memory phenotype. A Phase I clinical safety trial at Karolinska is currently prepared for IL-2/15/21-expanded TIL for the cellular therapy for patients with pancreatic cancer.

